# Duodenal quantitative mucosal morphometry in children with environmental enteric dysfunction: a cross-sectional multicountry analysis

**DOI:** 10.1016/j.ajcnut.2024.04.027

**Published:** 2024-04-27

**Authors:** Lubaina Ehsan, David Coomes, Paul Kelly, Adam R Greene, S Asad Ali, Chola Mulenga, Donna M Denno, Kelley VanBuskirk, Muhammad Faraz Raghib, Mustafa Mahfuz, Sean R Moore, Md Shabab Hossain, Tahmeed Ahmed, Peter B Sullivan, Christopher A Moskaluk, Sana Syed, EEDBI Consortium, Kumail Ahmed, Sheraz Ahmed, Md Ashraful Alam, SM Khodeza Nahar Begum, Subhasish Das, Lee A Denson, Shah Mohammad Fahim, Md Amran Gazi, Md Mehedi Hasan, Aneeta Hotwani, Junaid Iqbal, Najeeha Talat Iqbal, Zehra Jamil, Furqan Kabir, Ta-Chiang Liu, Ramendra Nath Mazumder, Shyam S Ragahavan, Masudur Rahman, Najeeb Rahman, Kamran Sadiq, Shafiqul Alam Sarker, Phillip I Tarr, Guillermo J Tearney, Fayaz Umrani, Grace Umutesi, Omer H Yilmaz

**Affiliations:** 1 Division of Pediatric Gastroenterology, Hepatology, and Nutrition, Department of Pediatrics, University of Virginia, Charlottesville, VA, United States; 2 Department of Epidemiology, University of Washington, Seattle, WA, United States; 3 Blizard Institute, Barts and The London School of Medicine, Queen Mary University of London, London, United Kingdom; 4 Department of Paediatrics and Child Health, Aga Khan University, Karachi, Pakistan; 5 Tropical Gastroenterology and Nutrition Group, University of Zambia School of Medicine, Lusaka, Zambia; 6 Department of Pediatrics, University of Washington, Seattle, WA, United States; 7 Department of Global Health, University of Washington, Seattle, WA, United States; 8 Department of Pediatrics, University of Virginia, Charlottesville, VA, United States; 9 Nutrition Research Division, International Centre for Diarrhoeal Disease Research, Bangladesh, Dhaka, Bangladesh; 10 Department of Paediatrics, Children’s Hospital, University of Oxford, Oxford, United Kingdom; 11 Department of Pathology, University of Virginia, Charlottesville, VA, United States

**Keywords:** pediatric, global health, gastrointestinal morphology, villi, crypt

## Abstract

**Background:**

Environmental enteric dysfunction (EED), a chronic inflammatory condition of the small intestine, is an important driver of childhood malnutrition globally. Quantifying intestinal morphology in EED allows for exploration of its association with functional and disease outcomes.

**Objectives:**

We sought to define morphometric characteristics of childhood EED and determine whether morphology features were associated with disease pathophysiology.

**Methods:**

Morphometric measurements and histology were assessed on duodenal biopsy slides for this cross-sectional study from children with EED in Bangladesh, Pakistan, and Zambia (*n* = 69), and those with no pathologic abnormality (NPA; *n* = 8) or celiac disease (*n* = 18) in North America. Immunohistochemistry was also conducted on 46, 8, and 18 biopsy slides, respectively. Linear mixed-effects regression models were used to reveal morphometric differences between EED compared with NPA or celiac disease and identify associations between morphometry and histology or immunohistochemistry among children with EED.

**Results:**

In duodenal biopsies, median EED villus height (248 μm), crypt depth (299 μm), and villus:crypt (V:C) ratio (0.9) values ranged between those of NPA (396 μm villus height; 246 μm crypt depth; 1.6 V:C ratio) and celiac disease (208 μm villus height; 365 μm crypt depth; 0.5 V:C ratio). Among EED biopsy slides, morphometric assessments were not associated with histologic parameters or immunohistochemical markers, other than pathologist-determined subjective semiquantitative villus architecture.

**Conclusions:**

Morphometric analysis of duodenal biopsy slides across geographies identified morphologic features of EED, specifically short villi, elongated crypts, and a smaller V:C ratio relative to NPA slides, although not as severe as in celiac slides. Morphometry did not explain other EED features, suggesting that EED histopathologic processes may be operating independently of morphology. Although acknowledging the challenges with obtaining relevant tissue, these data form the basis for further assessments of the role of morphometry in EED.

## Introduction

Environmental enteric dysfunction (EED) is an asymptomatic acquired disorder characterized by persistent inflammation of the small intestine, epithelial permeability, and villus blunting [1]. EED is an important cause of childhood undernutrition in low- and middle-income countries due to associated malabsorption and chronic systemic inflammation (by translocation of microbial products and/or enteric inflammation) [2,3]. EED causes undernutrition refractory to nutritional interventions, which is associated with other deleterious health impacts in childhood and later-adult years, such as delayed neurocognitive development [[4], [5], [6]]. Although the etiology of EED is not well understood, it is highly prevalent in low-resource settings with poor sanitation and hygiene and likely driven by exposures to an environment highly contaminated with fecal microbes.

Similar to EED, untreated celiac disease interferes with nutrient absorption, leading to undernutrition and poor growth [7,8]. In 2020, ∼49 million children aged <5 years worldwide (22%) had faltered linear growth [9], making improved scientific understanding and clinical interventions for malabsorptive enteropathies an urgent global need.

EED is currently diagnosed via endoscopically acquired biopsies, with a few noninvasive biomarkers (none specific to EED), and no definitive treatment [1,10,11]. A companion paper in this issue provides a pathology scoring protocol to histologically identify EED and disease severity [12]. Numerous financial, ethical, and operational challenges make obtaining small bowel biopsies from children challenging, whereas sampling biopsies from well-growing children includes additional ethical challenges [13]. As such, only a few studies have investigated the mucosal features of EED, especially in relationship to comparison groups. The EED Biopsy Initiative (EEDBI) Consortium, comprising 3 studies, all with the shared goal of advancing the understanding of EED pathophysiology, offers a unique opportunity to assess duodenal biopsies from children with EED and children with celiac disease and no clinical pathology.

Although intestinal architecture can be subjectively categorized by histologic assessment, morphometry provides a quantitative method for measuring mucosal morphology which can subsequently be correlated with both functional and disease outcomes. In this study, we sought to further improve our understanding of EED pathogenesis by associating villus and crypt morphometry measurements with key histologic markers of disease. Comparing morphometric features in the duodenum of well-characterized children with EED to comparison groups of North American children with celiac disease or those with no pathologic abnormalities (NPA) allowed us to define the morphometric characteristics of EED as well as measure the impact of EED on histologic architecture. Finally, we investigated EED morphometry association with histologic features and immunohistochemical markers of disease.

## Methods

### Cohorts and biopsy acquisition

The individual studies included in this analysis have been described in detail elsewhere, including recruitment and sampling timeframes, enrollment procedures, eligibility criteria, nutritional management, and study participant characteristics [14]. Upper gastrointestinal (GI) endoscopy, biopsy, slide preparation, histology scoring, and immunohistochemistry methods have also been described in detail elsewhere [12,15]. In brief, children with undernutrition unresponsive to nutritional intervention and no identifiable etiology for their growth faltering were considered to have EED and were enrolled for upper GI endoscopy with duodenal pinch forceps biopsy at 3 study centers: Aga Khan University, Karachi, Pakistan; the International Centre for Diarrhoeal Disease Research, Dhaka, Bangladesh; and the University Teaching Hospital, Lusaka, Zambia. These study centers enrolled children residing in low-income communities, 2 of which were urban (Dhaka, Bangladesh and Lusaka, Zambia) and 1 was rural (Matiari, Pakistan). Endoscopy exclusion criteria across the 3 centers included a recent history of diarrhea, laboratory findings consistent with a risk factor for an underlying bleeding disorder, and a medically identifiable etiology of refractory undernutrition, including tissue transglutaminase IgA concentrations suggestive of celiac disease.

Comparison groups of children undergoing upper GI endoscopy with duodenal pinch forceps biopsy for clinical indications were enrolled at Cincinnati Children’s Hospital Medical Center (CCHMC, Cincinnati, United States). Biopsies determined to have no pathologic abnormality (NPA) or celiac disease by a clinical pathologist were included in this study. Children with evidence of eosinophilic esophagitis or gastric or duodenal histologic abnormalities were excluded from the NPA group. Biopsy samples were collected across studies between November 2016 to August 2019; details regarding recruitment and other timeframes are presented elsewhere [14].

### Morphometry, histology, and immunohistochemistry

Duodenal mucosal biopsies were fixed in formalin, paraffin-embedded, sectioned, and stained with hematoxylin and eosin (H&E); similar standard operating procedures were used for histology processing across all centers. Stained biopsy glass slides from Pakistan, Bangladesh, and Zambia were digitized using the Olympus VS120 scanner (Olympus Corporation Inc.). The Olympus VS120 scanner and the Leica SCN400 brightfield scanner (Leica Microsystems CMS GmbH) were used for celiac disease and NPA comparison slides from CCHMC. All glass slides were scanned at ×40 magnification. The same scanned biopsy slides were utilized for morphometry and semiquantitative scoring.

Olympus CellSens dimension was used for biopsies digitized via Olympus VS120 (vsi image file format; Pakistan, Bangladesh, Zambia, and a subset from CCHMC) and Aperio ImageScope for those digitized via the Leica SCN400 brightfield scanner (svs image file format; the remaining CCHMC slides). A biopsy slide can have multiple biopsy sections so a section without preparation artifacts such as clumped stains or bubbles was selected for morphometry. Morphometric analysis was done via Olympus CellSens dimension and Aperio ImageScope software. From each biopsy image, a maximum of 3 villus and crypt units were independently measured by 2 research assistants (LE, MFR) and reviewed by 2 pediatric gastroenterologists (SS, PBS) and a GI pathologist (CAM). Labeling of slide images precluded researcher blinding of slide assignment to study cohort.

**FIGURE 1:**
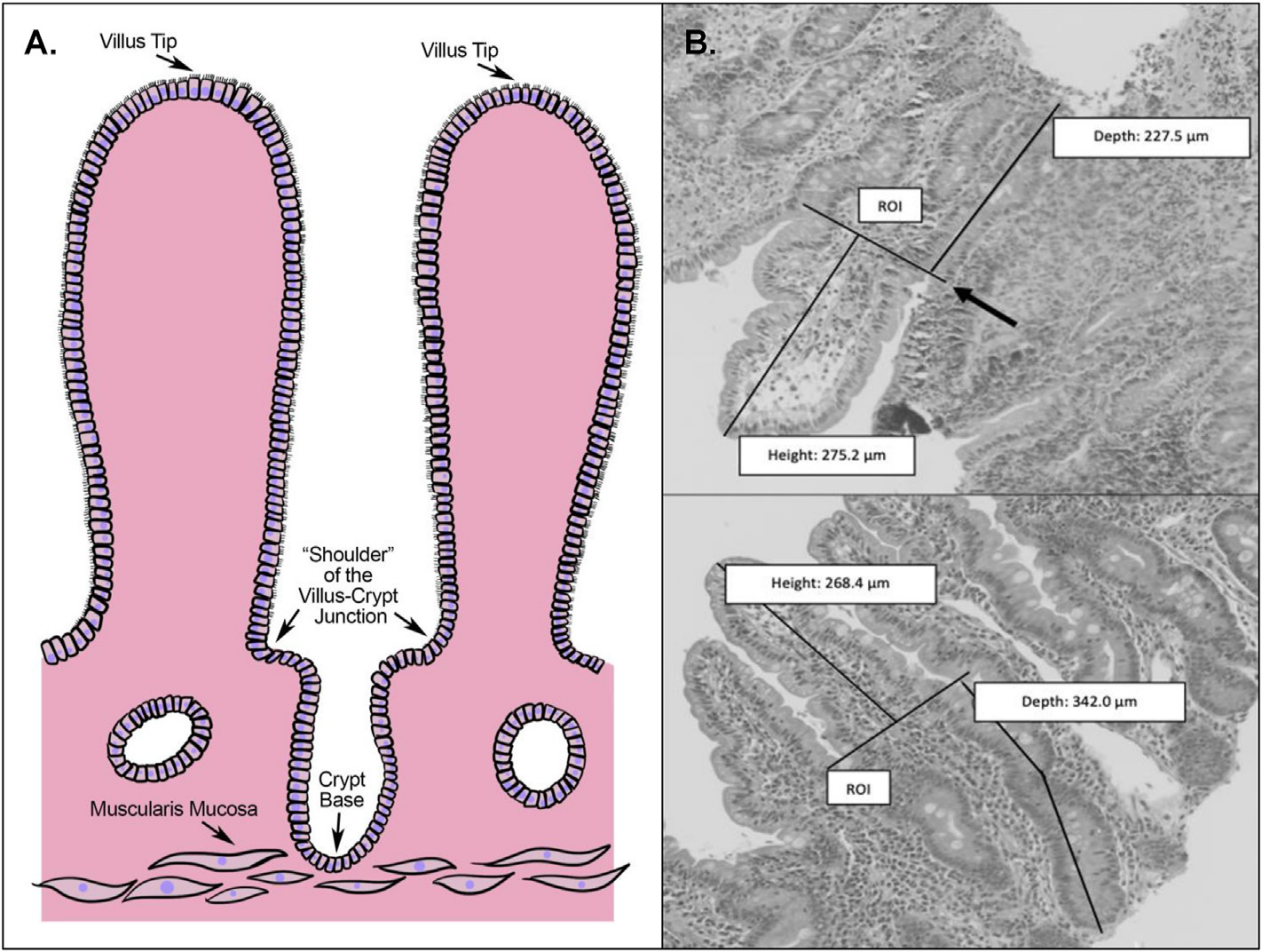
Villus length and crypt depth morphometry measurements from duodenal biopsy images. (A) Illustration of villus and crypt structures that were identified for morphometric measurements. (B) Mucosal morphometry was measured from villus–crypt units present in individual-derived duodenal biopsy tissue. Top image: Villus length was measured from the tip of the villi to the “shoulder” of the villus in the crypt–villus junction, marking the start of the crypt (height). Crypt depth was measured from the “shoulder” of the crypt–villus junction to the base of the crypt (depth). Arrow: the region of interest (ROI) for visualizing the villus–crypt junction, or the end of the mucosa or beginning of the submucosa, which enables the assessment of the depth of the mucosa. Bottom image: Morphometric measurement where crypt depth was measured taking the curvature of the crypt into account.

Morphometric measurements were conducted on slides where the majority of the villus–crypt axes were sufficiently visible to enable linear measurement. Villus length was measured from the tip of the villus to the “shoulder” of the villus–crypt junction at the beginning of the submucosa, where there was a scaffold in the epithelium marking the start of the crypt. Crypt depth was measured from the “shoulder” of the villus–crypt junction to the base of the crypt. The crypt base was marked where the muscularis mucosa or beginning of submucosa was visualized (Figure 1). For crypt depth measurements where the muscularis mucosa or start of submucosa was visible without the complete crypts being evident, we extended the measurement from the villus–crypt junction to the muscularis mucosa or start of submucosa to reduce the risk of underestimating crypts in the absence of suitable sectioning. The average of 2 measurements for each crypt, villus, and V:C ratio was computed and reported.

H&E-stained slides were semiquantitatively scored by 2 or 3 gastrointestinal pathologists using a standardized EED scoring criteria consisting of 8 parameters, as described in detail elsewhere [11] and in this series [12]. Our analysis focused on 3 representative parameters – villus architecture score, Paneth cell depletion score, and goblet cell depletion score – which were a priori considered to be potentially related to villus height, crypt depth, and V:C ratio. Paneth cells secrete factors that help sustain and modulate the epithelial stem and progenitor cells that cohabitate in the crypts and rejuvenate the small intestinal epithelium, whereas goblet cells are intestinal mucosal epithelial cells that synthesize and secrete mucus. The metrics for scoring these parameters are detailed in Table 1. A more detailed analysis of these observations can be found elsewhere [12].

**Table 1:** Histology scoring metrics for environmental enteric dysfunction.

Score^1^	Scoring metrics
Villus architecture score	
0	Majority of villi are >3 crypt lengths long.
1	Majority of villi are ≤3 crypt lengths long, but >1 crypt length long. Involves <50% of the mucosa.
2	Majority of villi are ≤3 crypt lengths long, but >1 crypt length long. Involves >50% of the mucosa.
3	Mostly absent villi, or majority of villi are ≤1 crypt length long. Involves <50% of the mucosa.
4	Mostly absent villi or a majority of villi are ≤1 crypt length long. Involves >50% of the mucosa.
Paneth cell depletion	
0	≥5 Paneth cells/crypt, on average.
1	2–4 Paneth cells/crypt, on average.
2	<2 Paneth cells/crypt, involving <50% of crypt bases.
3	≥5 Paneth cells/crypt, involving >50% of crypt bases.
Goblet cell depletion	
0	Normal goblet cell density (≥1 goblet cell per 20 enterocytes) in all evaluable mucosal epithelium.
1	Decreased goblet cells (<1 per 20 enterocytes) in 1%–25% of evaluable mucosal epithelium.
2	Decreased goblet cells (<1 per 20 enterocytes) in 26%–50% of evaluable mucosal epithelium.
3	Decreased goblet cells (<1 per 20 enterocytes) in 51%–75% of evaluable mucosal epithelium.
4	Decreased goblet cells (<1 per 20 enterocytes) in 76%–100% of evaluable mucosal epithelium.

1 For all parameters, lower values represent less injury and higher values represent more injury.

Tissue microarrays from a subset of the paraffin-embedded blocks from the EEDBI cohorts were created and histologically sectioned at the University of Virginia. Due to clinical restrictions, blocks could not be released from CCHMC, so histologic sections were sent to University of Virginia where all sections were then stained by immunohistochemistry (IHC) using panels of antibodies, and digitally imaged and quantitated, as described in a companion paper [15]. The IHC antibodies for this analysis were selected to reflect molecular targets that would accompany villus injury and crypt elongation repair responses (Supplemental Table 1). Notably, some IHC measurements were lacking among some participants with celiac disease due to insufficient samples for complete IHC panel measurements.

Slides with only IHC or histology measurements and duplicate slides from the same individual were excluded. In the case of duplicate slides, the slide with more morphometric measurements was included. The morphometry comparison with IHC readouts and histology scores was only conducted on slide images from the paired identical paraffin blocks. Other than for 5 individuals for whom biopsies from the first segment of the duodenum were analyzed, biopsies from the second/third segments of the duodenum were utilized in this analysis.

### Statistical analysis

Linear mixed-effect regression models were used to estimate morphometric associations with other measures of interest. Each model included 1 morphometric measurement (villous height, crypt depth, or V:C ratio) and 1 independent fixed-effect variable [disease classification (EED, celiac, or NPA), histology parameter, or IHC variable] and included a random intercept for each individual. Mixed-effects models account for the nonindependence of multiple morphometric measurements per individual. Morphometry and IHC measurements were log-transformed to reduce the influence of outliers.

One set of models was used to test for morphometry differences according to disease classification (EED, NPA, or celiac), with EED as the comparison group. Another set of models tested the association of morphometry with selected histology parameters and IHC markers among individuals with EED.

Inclusion of gender as a covariate across morphometry models did not result in meaningful (>10%) difference in point estimates. Age was colinear with disease status (i.e., there was no overlap between the EED cohorts with the celiac or NPA cohorts), limiting our ability to adjust for age. Adjusting for age did not impact the point estimates of histologic differences between EED and the comparison groups presented in a companion paper in this issue [12]. Hence only unadjusted models are presented for morphometry-disease status models. Age across the EED cohorts was similar; however, for a few morphometry-IHC models, the addition of age as a covariate shifted a few point estimate by >10%, although without influence on statistical significance. Hence, in EED-only models, we presented multivariable results adjusted for EEDBI center, and as supplemental material, we also presented models adjusted for EEDBI center and age. Analyses were performed with R (version 4.2.1).

### Ethical approvals

Signed informed consent from parents or legal guardians was obtained and all study centers obtained approvals from their respective review boards. Details on these procedures can be found elsewhere in this supplemental issue [14].

## Results

### Characteristics of the EED cohorts

**FIGURE 2:**
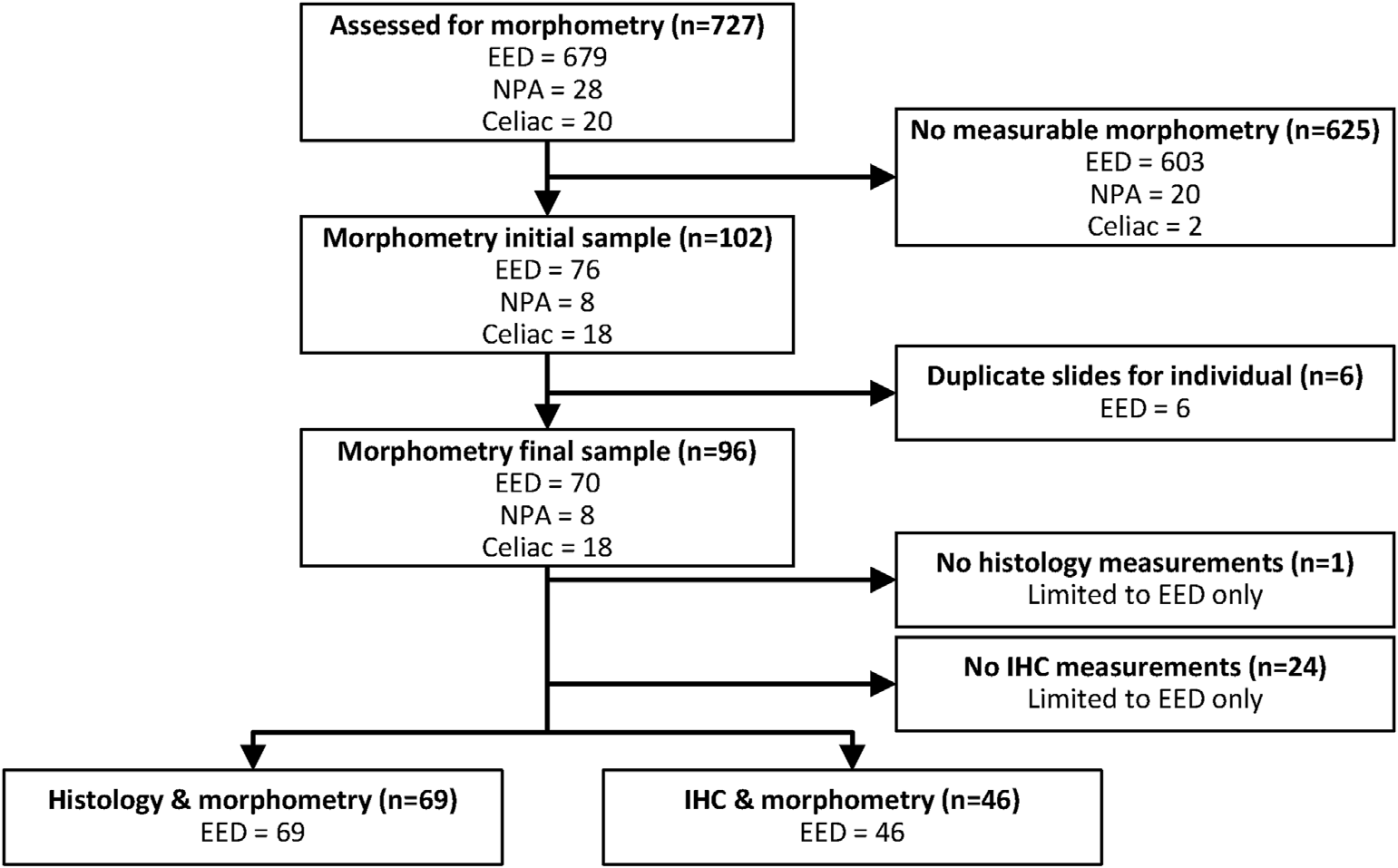
Flowchart depiction of the biopsy slides obtained and used within this study. A total of 727 duodenal biopsy slides were obtained from children enrolled at 4 EEDBI Consortium centers (BEECH, Biomarkers of Environmental Enteropathy in Children, University Teaching Hospital, Zambia; BEED, Bangladesh Environmental Enteric Dysfunction, International Centre for Diarrhoeal Disease Research, Bangladesh; SEEM, Study of Environmental Enteropathy and Malnutrition, Aga Khan University, Pakistan; CCHMC, Cincinnati Children's Hospital Medical Center, Cincinnati, United States). Slides where no morphometric measurements were obtained and duplicate slides per individual were excluded from our analysis. Environmental enteric dysfunction (EED) slides were further analyzed for histology, morphometry, and immunohistochemistry.

291 children with EED were enrolled at 3 different EEDBI centers: the Biomarkers of Environmental Enteropathy in Children (BEECH) study in Zambia (*n* = 108 with 205 biopsy slides), the Bangladesh Environmental Enteric Dysfunction (BEED) study (*n* = 120 with 240 biopsy slides), and the Study of Environmental Enteropathy and Malnutrition study in Pakistan (*n* = 63 with 234 biopsy slides) (Figure 2). Comparison groups of children, each with one biopsy slide, with NPA (*n* = 28) and celiac disease (*n* = 20) were enrolled at CCHMC in Cincinnati, OH. After excluding slides without morphometric measurements (*n* = 603 EED, *n* = 20 NPA, and *n* = 2 celiac), our analysis was limited to 102 slides with morphometric measurements (*n* = 76 EED, *n* = 8 NPA, and *n* = 18 celiac). Six duplicate slides from the same individual were excluded; the slide with more morphometric measurements was retained. Our final data set for morphometric analysis consisted of 96 biopsy slides from 96 individual children (*n* = 70 EED, *n* = 8 NPA, and *n* = 18 celiac). Among the 70 children with EED and morphometric measurements, 69 had paired histology scores and 46 had paired IHC measurements (Table 2).

**Table 2:** Demographic and anthropometric characteristics, by cohort.

Variable	BEECH	BEED	SEEM	EED combined	CCHMC NPA	CCHMC Celiac
Observations with morphometric measurements (# Children/#Measurements)	20/35	32/51	18/48	70/134	8/22	18/46
Age (mo) [median; (IQR)]	18.6 (16.3, 20.9)	19.0 (17.5, 20.6)	17.0 (14.6, 21.8)	18.7 (16.3, 21.2)	80.8 (65.9, 85.3)	99.3 (76.6, 127.4)
Gender [# Female; (%)]	9 (45.0)	17 (53.1)	4 (22.2)	30 (42.9)	6 (75.0)	10 (55.6)
HAZ/LAZ [median; (IQR)]	−3.3 (−3.7, −2.9)	−2.3 (−3.0, −1.6)	−3.2 (−3.7, −2.4)	−2.9 (−3.5, −2.1)	1.0 (0.5, 1.6)	−0.2 (−0.8, 0.7)
WAZ [median; (IQR)]	−2.2 (−2.5, −1.9)	−1.8 (−2.6, −1.3)	−2.8 (−3.5, −2.6)	−2.3 (−2.8, −1.7)	1.4 (0.8, 2.0)	0.1 (−1.0, 0.4)
WHZ/WLZ [median; (IQR)]	−0.8 (−1.2, −0.5)	−1.0 (−1.7, −0.5)	−1.8 (−2.6, −1.4)	−1.1 (−1.8, −0.6)	1.3 (0.7, 1.8)	0.2 (−0.2, 0.6)
BMI [median; (IQR)]	14.7 (14.6, 14.8)	N/A	N/A	14.7 (14.6, 14.8)	16.7 (15.2, 18.7)	15.5 (14.8, 16.5)
Samples with paired morphometry and histology data (# Children/# Measurements)	19/33	32/51	18/48	69/132	3/8	16/40
Samples with paired morphometry and IHC data (# Children/# Measurements)	7/13	28/46	11/28	46/87	2/5	9/26

Abbreviations: BEECH, Biomarkers of Environmental Enteropathy in Children, University Teaching Hospital, Zambia; BEED, Bangladesh Environmental Enteric Dysfunction, International Centre for Diarrhoeal Disease Research, Bangladesh (icddr,b); CCHMC, Cincinnati Children's Hospital Medical Center, Cincinnati, United States; HAZ, height-for-age *z*-score; IQR, interquartile range; LAZ, length-for-age *z*-score; N/A, not available; NPA, no pathologic abnormality; SD, standard deviation; SEEM, Study of Environmental Enteropathy and Malnutrition, Aga Khan University, Pakistan; WAZ, weight-for-age *z*-score; WHZ, weight-for-height *z*-score, WLZ, weight-for-age *z*-score.

The median age of children with EED was 19 mo (19 mo in BEECH, 19 mo in BEED, and 17 mo in Study of Environmental Enteropathy and Malnutrition), 81 mo for the NPA cohort, and 99 mo for children with celiac disease. This age difference is not surprising because children aged <2 y rarely have clinical indications for upper endoscopies resulting in a celiac or NPA outcome. Anthropometric measurements (height-for-age, weight-for-age, and weight-for-height *z*-scores) were substantially lower in the EED cohort compared with the others (Table 2). The demographic and anthropometric measurements of the cohort in this analysis here are similar to those of the full EEDBI Consortium cohort [14].

### Quantitative mucosal morphometry measurements of duodenal biopsy tissue

**FIGURE 3:**
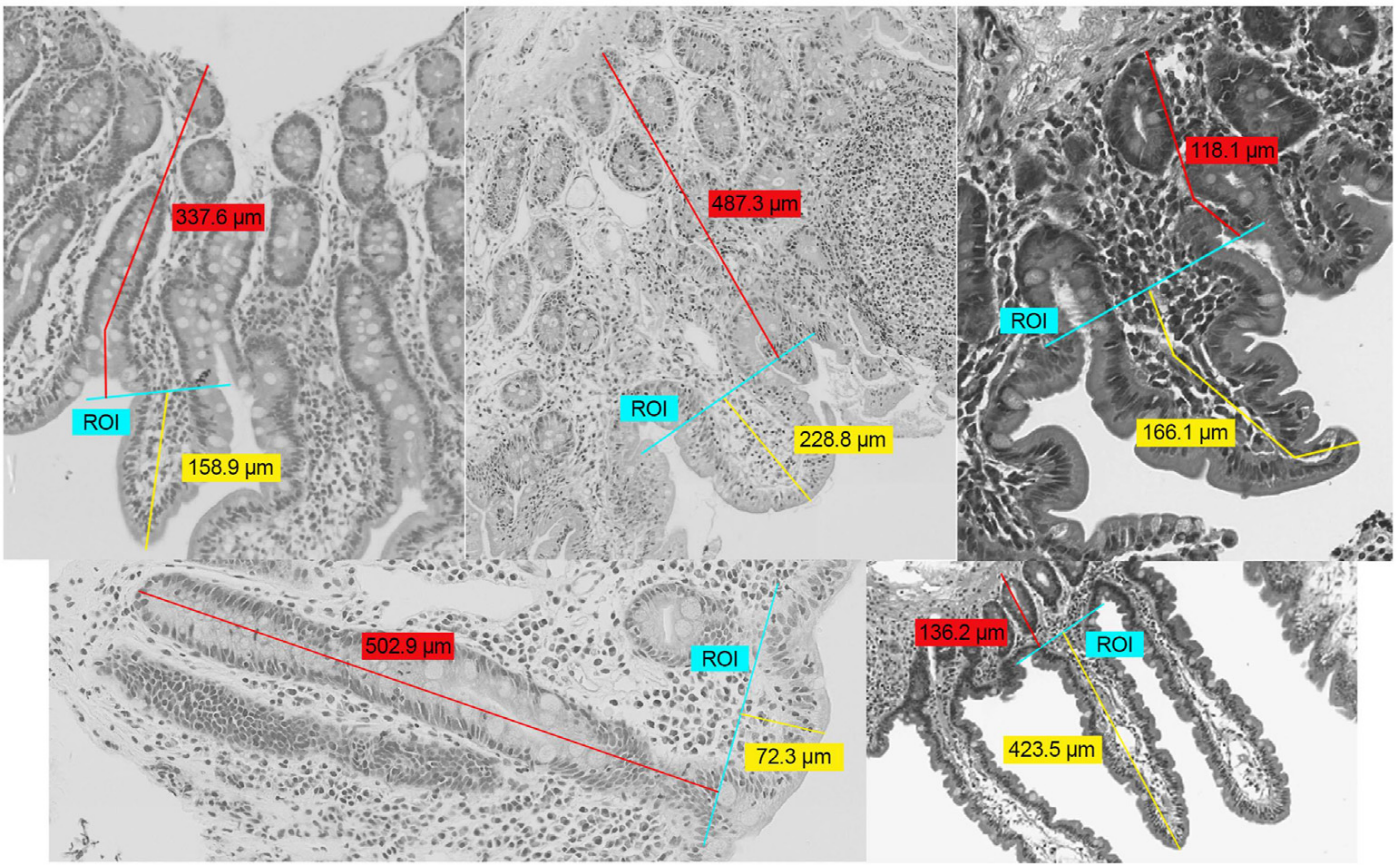
Morphometric measurement examples from across sites. Top row right to left: SEEM, BEED, and BEECH. Bottom row right to left: CCHMC Celiac and CCHMC NPA. Measurements within red boxes indicate crypt depth whereas those within yellow boxes indicate villus height. Region of interest (ROI, as described in methods) has been drawn in blue for visualizing the villus–crypt junction. Complete crypt depth was not evident for SEEM, BEED, BEECH, and NPA as shown in the images due to which measurements were extended to the muscularis mucosa as complete crypt depth was not evident to reduce the risk of crypt underestimation, as detailed in the methods section. Abbreviations: BEECH, Biomarkers of Environmental Enteropathy in Children, University Teaching Hospital, Zambia; BEED, Bangladesh Environmental Enteric Dysfunction, International Centre for Diarrhoeal Disease Research, Bangladesh (icddr,b); CCHMC, Cincinnati Children's Hospital Medical Center, Cincinnati, United States; NPA, no pathologic abnormality; SEEM, Study of Environmental Enteropathy and Malnutrition, Aga Khan University, Pakistan.

Biopsies from children with EED were found to have shorter median villi compared with those with NPA but longer villi than those with celiac disease (EED = 248 μm, NPA = 396 μm, and celiac = 208 μm; Table 3). Conversely, deeper median crypts were measured in EED biopsies compared with those from the NPA group, but crypts were shallower compared with celiac biopsies (EED = 299 μm, NPA = 246 μm, and celiac = 365 μm; Table 3). The median V:C ratios for EED biopsies were lower than NPA and higher than celiac disease (EED = 0.89, NPA = 1.59, and celiac = 0.52; Table 3). Mixed-effects regression models comparing morphometry between disease groups demonstrated NPA villi were 51% (95% confidence interval [CI]: 15%, 99%) longer and crypts were 20% (95% CI: 32%, 4%) shallower than EED biopsies (Table 4). Celiac biopsy villi were 25% (95% CI: 39%, 8%) shorter and crypts were 21% (95% CI: 7%, 37%) deeper than EED biopsies (Table 4). Examples of morphometric measurements from across sites are shown in Figure 3.

**Table 3:** Descriptive statistics [median and (interquartile range)] showing morphometric measurements, histology scores, and immunohistochemistry readouts among EEDBI cohorts and North American comparison groups.

Measurement	Normalization^1^	BEECH 35	BEED 51	SEEM 48	EED totals 134	CCHMC NPA 22	CCHMC Celiac 46	*N* (%) Missing^2^
Morphometry								
Villus height (μm)	N/A	255 (182, 307)	247 (207, 310)	248 (206, 324)	248 (206, 315)	396 (355, 430)	208 (121, 255)	N/A
Crypt depth (μm)	N/A	232 (182, 276)	286 (243, 348)	335 (306, 389)	299 (238, 355)	246 (189, 263)	365 (292, 422)	N/A
V:C ratio	N/A	1.1 (0.8, 1.5)	0.9 (0.6, 1.2)	0.7 (0.6, 1.2)	0.9 (0.6, 1.2)	1.6 (1.4, 2.0)	0.5 (0.3, 1.0)	N/A
Histology								
Villus Architecture Score	N/A	2.5 (1.1, 3.0)	2.0 (1.0, 3.0)	1.5 (0.3, 3.0)	2.0 (1.0, 3.0)	0.5 (0.3, 0.5)	3.8 (2.5, 4.0)	29 (14.4%)
Paneth cell depletion score	N/A	2.0 (1.0, 3.0)	2.0 (1.0, 3.0)	0.5 (0.4, 0.9)	1.0 (0.6, 3.0)	0.0 (0.0, 0.3)	0.5 (0.5, 1.0)	36 (17.8%)
Goblet cell depletion score	N/A	1.5 (1.0, 2.0)	2.0 (1.5, 2.5)	0.9 (0.5, 1.0)	1.5 (1.0, 2.0)	0.0 (0.0, 0.3)	0.5 (0.5, 1.0)	22 (10.9%)
Immunohistochemistry^3^								
IEL area	Epithelial area	76.9 (26.4, 89.7)	62.9 (40.3, 90.7)	123.2 (111.3, 133.3)	89.7 (49.9, 115.3)	119.2 (42.1, 119.2)	106.0 (87.7, 124.2)	84 (41.6%)
IEL area	Surface area	25.8 (11.8, 44.4)	37.7 (27.5, 57.9)	65.6 (52.2, 72.0)	44.4 (26.6, 63.2)	71.4 (21.7, 71.4)	47.8 (42.1, 51.7)	84 (41.6%)
IEL count	Epithelial area	0.22 (0.11, 0.29)	0.21 (0.14, 0.25)	0.23 (0.21, 0.33)	0.21 (0.17, 0.29)	0.25 (0.13, 0.25)	0.22 (0.19, 0.23)	84 (41.6%)
IEL count	Surface area	0.09 (0.08, 0.14)	0.12 (0.09, 0.16)	0.12 (0.12, 0.16)	0.12 (0.09, 0.16)	0.15 (0.07, 0.15)	0.09 (0.09, 0.10)	41.6
REG1B area	Surface area	5.8 (5.4, 17.7)	3.2 (0.8, 7.9)	4.5 (3.9, 10.3)	4.3 (1.3, 9.8)	0.7 (0.7, 0.7)	No observations	112 (55.4%)
MKI67 area	Epithelial area	346 (255, 426)	138 (111, 203)	372 (338, 519)	217 (123, 329)	301 (301, 301)	No observations	114 (56.4%)
Paneth cell (Defensin 5) area	Epithelial area	55.8 (39.6, 97.1)	29.1 (16.9, 45.8)	55.5 (45.8, 76.1)	39.3 (21.8, 55.8)	53.7 (48.5, 58.8)	20.8 (18.5, 66.4)	84 (41.6%)
Paneth cell (Defensin 5) area	Surface area	38.1 (18.7, 46.5)	17.9 (8.9, 27.1)	29. 9 (22.8, 36.0)	21.3 (13.8, 31.9)	29.5 (27.7, 31.2)	10. 5 (9.2, 30.9)	84 (41.6%)
SLC15A1	Surface area	67.4 (16.2, 76.6)	110.5 (92.2, 142.6)	217.6 (157.3, 240.9)	117.8 (92.2, 170.3)	170.2 (170.2, 170.2)	No observations	118 (58.4%)
SLC15A1-b	SLC15A1-c	245 (166, 306)	261 (204, 357)	197 (118, 426)	248 (170, 361)	735 (735, 735)	No observations	121 (59.9%)
DUOX2 area	Surface area	1.5 (0.8, 1.7)	1.8 (0.7, 4.4)	0.1 (0.0, 0.3)	0.7 (0.1, 2.0)	0.006 (0.006, 0.012)	0.03 (0.02, 0.09)	83 (41.1%)
Sucrase isomaltase area	Surface area	52.7 (40.8, 104.9)	65.4 (52.2, 100.8)	84.3 (77.6, 98.7)	77.6 (53.7, 101.5)	95.9 (58.7, 95.9)	48.6 (33.9, 79.4)	84 (41.6%)

Abbreviations: BEECH, Biomarkers of Environmental Enteropathy in Children, University Teaching Hospital, Zambia; BEED, Bangladesh Environmental Enteric Dysfunction, International Centre for Diarrhoeal Disease Research, Bangladesh (icddr,b); CCHMC, Cincinnati Children's Hospital Medical Center, Cincinnati, United States; EA, epithelial area; EED, environmental enteric dysfunction; IEL, intraepithelial lymphocyte; IQR, interquartile range; N/A, not available; NPA, no pathologic abnormality; SA, surface; SEEM, Study of Environmental Enteropathy and Malnutrition, Aga Khan University, Pakistan; SLC15A1, Solute Carrier Family 15 Member 1; SD, standard deviation; V:C ratio, villus:crypt ratio.

1 Because the biopsies varied in size, we normalized IHC measurements to tissue area depending on IHC function: EA, SA, SLC15A1-c (epithelial cytoplasm).

2 Refers to the number and percentage of morphometry measurements in our analysis that did not have paired histology scores or immunohistochemistry readouts from the same paraffin block.

3 Area measurements are in μm^2^. Immunohistochemistry measurements were multiplied by ×1000 for ease of interpretation.

**Table 4:** Linear mixed-effects model estimates comparing morphometric measurements between EED with NPA and celiac disease.

Disease state compared with EED^1^	% Difference (95% CI)^2^
NPA	
Villus height	51 (15, 99)
Crypt depth	−20 (−32, −4)
V:C ratio	89 (35, 165)
Celiac	
Villus height	−25 (−39, −8)
Crypt depth	21 (7, 37)
V:C ratio	−38 (−52, −20)

Abbreviations: CI, confidence interval; EED, environmental enteric dysfunction; NPA, no pathologic abnormality; V:C ratio, villus:crypt ratio.

1 *n* 98 individuals, 207 morphometric measurements.

2 Measurements were log-transformed and reported as the mean % difference (with 95% CI) in morphometry variable point estimates normalized to EED. For example: 51 can be interpreted as 51% greater villus height for NPA biopsies compared with EED biopsies.

### Morphometric measurement associations with histology scores among children with EED

We next explored whether morphometric measurements in EED biopsies are associated with 3 specific histologic parameters: villus architecture score, Paneth cell depletion score, and goblet cell depletion score. Univariate and multivariable models adjusted for EEDBI center are presented in Table 5, and multivariable models additionally adjusted for age are reported in Supplemental Table 2. Median scores for the histologic parameters are presented in Table 3.

**TABLE 5:** Linear mixed-effects model estimates associating morphometric measurements with histology scores among participants with EED; multivariable results are adjusted for the EEDBI center.

Morphometry – histology scores	*n*	Univariate model % Difference(95% CI)	Multivariable model % Difference(95% CI)
Villus height – Villus architecture	64	−14 (−19, −8)	−13 (−19, −7)
Villus height – Paneth cell depletion	64	−3 (−11, 5)	−2 (−11, 9)
Villus height – Goblet cell depletion	58	−4 (−14, 8)	−2 (−14, 11)
Crypt Depth – Paneth cell depletion	69	−8 (−13, −2)	−4 (−10, 2)
V:C ratio – Villus architecture	58	−16 (−23, −9)	−18 (−24, −12)

Morphometric measurements were log-transformed, whereas histology scores were not. Point estimates are reported as the mean % difference in the morphometry variable per each unit higher histology score. For example: −14 can be interpreted as each 1-unit higher villus architecture score is associated with 14% lower villus height.

Abbreviations: CI, confidence interval; V:C ratio, villus:crypt ratio.

Higher pathologist-scored villus architecture (indicating more severe villus blunting) (Table 1) was significantly associated with 13% (95% CI: 7%, 19%) lower villus height and 18% (95% CI: 12%, 24%) lower V:C ratio, after adjusting for EEDBI study center. Although higher Paneth cell depletion scores were associated with lower crypt depth in unadjusted models, this association was not maintained in the multivariable model. Goblet cell depletion was not associated with morphometry.

### Association of EED morphometry with immunohistochemistry markers

Next, we sought to determine whether morphometric measurements in EED tissue were associated with specific IHC markers. Median IHC measurements are included in Table 3. A detailed analysis of these IHC findings are discussed elsewhere [15]. Of the 11 IHC antibodies tested, only Solute Carrier Family 15 Member 1 (SLC15A1) and Regenerating Family Member 1 Beta (REG1B) were significantly associated with morphometric measurements in univariate models (Table 6). SLC15A1 is a hydrogen peptide cotransporter commonly used as a functional marker of the epithelial brush border. REG1B is a common cytoplasmic marker of epithelial repair that is commonly expressed in the colonic and small intestinal epithelium. The REG1B–crypt depth association neared statistical significance after adjusting for the EEDBI study center, and the SLC151–villus height association was attenuated and lost significance in the multivariable model (Table 6 and Supplemental Table 3).

**TABLE 6:** Linear mixed-effects model estimates associating morphometric measurements with immunohistochemistry readouts in EED cohorts, adjusted for EEDBI center.

Morphometry – IHC variables	Normalization	N	Univariate model	Multivariable model
			% Difference (95% CI)	% Difference (95% CI)
Villus height (dependent)				
Lymphocyte infiltration (IEL area)	EA	46	0.9 (−0.8, 2.7)	0.3 (−1.7, 2.3)
Lymphocyte infiltration (IEL count)	EA	46	1.8 (−0.8, 4.4)	1.3 (−1.3, 4.0)
Epithelial brush border (SLC15A1 total)	SA	41	3.0 (1.0, 5.0)	2.8 (0.5, 5.1)
Epithelial brush border (SLC15A1-b)	SLC15A1-c	41	1.4 (−0.7, 3.6)	1.5 (−0.6, 3.5)
Epithelial cell cytoplasm (DUOX2)	SA	43	−0.3 (−0.9, 0.3)	−0.1 (−0.9, 0.7)
Brush border (sucrase isomaltase area)	EA	46	0.3 (−1.5, 2.1)	−0.1 (−1.9, 1.7)
Crypt depth (dependent)				
Lymphocyte infiltration (IEL area)	EA	46	0.8 (−0.2, 1.8)	0.3 (−0.8, 1.3)
Lymphocyte infiltration (IEL count)	EA	46	0.9 (−0.5, 2.4)	0.5 (−1.0, 1.9)
Epithelial repair (REG1B)	SA	46	−0.4 (−0.8, 0.0)	−0.4 (−0.8, −0.1)
Proliferating crypt cells (MKI67 area)	EA	44	0.6 (−0.4, 1.6)	0.3 (−1.0, 1.6)
Paneth cell area (defensin 5)	EA	46	−0.2 (−0.7, 0.4)	−0.3 (−0.8, 0.3)

Morphometryic measurements and IHC readouts were log-transformed. Coefficients are reported as the mean % difference in morphometry variable per 10% higher IHC variable. For example, 0.9 can be interpreted as every 10% higher lymphocyte area is associated with a 0.9% higher villus height.

Abbreviations: CI, confidence interval; EA, epithelial area; IEL, intraepithelial lymphocyte; IHC, immunohistochemistry; SA, surface area.

## Discussion

Our multicountry analysis demonstrates characteristic morphometric features of EED. Morphometry is a valuable technique to augment histologic analysis because it provides a method for objective quantitative measurements of biopsy morphology. Here, we found that villi in biopsies obtained from children with EED were 37% shorter, crypts were 22% deeper, and V:C ratios were 44% lower than NPA tissue.

Intestinal morphology data in healthy children have rarely been reported, due to ethical constraints in taking biopsies from children who are clinically well. One previous study among children from the United Kingdom undergoing jejunal biopsy for clinical indications found, among 22 with subjectively normal histology, a mean villus height of 332 μm, crypt depth of 169 μm, and V:C ratio of 2.0 [16]. We observed a somewhat higher median villus length (396 μm) in our NPA duodenal biopsies from children of similar age compared with the United Kingdom cohort and who also had subjectively normal histology (as determined by their clinical pathologist). However, biopsies in our NPA group had a median crypt depth that measured 246 μm; differences may be due to the relatively small number of children in the NPA cohort. Our findings in relation to duodenal morphology in children with celiac disease are what would be expected, namely, villus shortening and crypt hypertrophy of marked degree [17,18]. Values from our morphometric assessment of mucosal biopsies of children with EED lie between those considered normal and those with celiac disease. This suggests that the changes in EED are probably in response to pathologic processes at the luminal–mucosal interface.

EED biopsies measured in our study displayed noticeable villus shortening (248 μm), in agreement with previous findings published in a comprehensive study by Kelly, et al. [19] where the mean villus height shortening (265 μm) was noted among children from Zambia with enteropathy [20]. There have also been other previously published studies that describe villus shortening among children with enteropathy [20,21]. Furthermore, our EED biopsies displayed crypt elongation with a median depth of 299 μm. Crypt deepening has previously been characterized as a compensatory mechanism for villus atrophy [22], suggesting that the deeper crypts found in our EED biopsies may be an attempt to improve nutrient acquisition in response to the shortened villi. Crypt elongation among children with enteropathy is supported by previously published literature [20]. Together, these observations establish a baseline measurement of healthy intestinal architecture and demonstrate the substantial impacts of EED on duodenal morphology.

Villus height and the V:C ratio were inversely associated with the more subjective villus architecture score. This inverse relationship is to be expected and increases our confidence in the villus architecture parameter as a semiquantitative assessment of villus shortening. This villus architecture parameter is 1 of 5 histologic features recommended for assessment of biopsies to identify EED and quantify EED severity [12]. The villus architecture parameter, however, does not supplant information gleaned from quantitative morphometry and does not discriminate crypt depth from villus height.

The lack of association between morphometric measurements and other histology parameters or IHC antibodies could reflect independent processes at the mucosal surface. However, our lack of ability to find associations could also be due to several limitations of this study. First, only a select number of biopsies were sectioned with an orientation that allowed for accurate morphometric assessment. IHC staining was batched and conducted at a single center; however, differences in slide preparation and H&E staining between centers could have contributed to variability in histology assessment, potentially attenuating our ability to identify associations by pooling biopsies across study centers. In work presented in a companion paper in this issue (Kelly et al. [12]), differences in histologic features were noted between the 3 EEDBI centers, including for Paneth and goblet cell depletion, which were most prevalent in the BEED cohort. Variations in enrollment criteria could also have an influence. Furthermore, differences in histology between settings could be due to variations in underlying etiologies of EED, which are likely complex and multifaceted. Future investigations will be needed to parse out the cause and impact of histopathologic features of EED.

Our morphometry comparison of EED to NPA and celiac histology was also limited by our lack of within-country comparison groups, hence restricting the ability to identify regional baselines when investigating mechanisms of pathogenesis. To address this concern, many studies use cohorts of participants with other inflammatory diseases or comparison groups from higher resource settings where upper bowel biopsies are more commonly obtained for clinical purposes [15,20,23]. Furthermore, in EED-endemic regions, many individuals display significant amount of baseline inflammation, complicating the ability to assemble relevant comparison cohorts [20,24]. Another limitation to our study is the variation in age between EED and North American cohorts. Notably, children enrolled across the EEDBI centers were of similar age at the time of biopsy, and age was colinear with disease status when comparing EED to the North American cohorts. As adjusting for age did not impact the point estimates of our companion histology analysis [12], our results are likely due to physiological differences caused by EED, rather than age differences. Finally, we acknowledge the sample size limitations of this study. Numerous ethical and technical challenges impede biopsy tissue acquisition in young children [13]. However, Chandwe, et al. [25] have described the safety profile and clinical benefit for children with EED undergoing endoscopy, supporting its use to inform clinical research. We also acknowledge that from a morphometry standpoint, differences in absolute values of crypt depth measurement among the different EED sites may be due to a difference in the orientation of biopsies. Despite the aforementioned limitations, this study leverages data from the largest sample of EED biopsies among children to date.

In summary, morphometric analysis of duodenal biopsies has the potential to provide insight into the pathophysiology of EED and other nutritional diseases. Here, we characterize EED tissue as having significantly shortened villi and deeper crypts than tissue with NPA. Our findings add validity to the semiquantitative villus architecture parameter in the novel EED histology scoring system [12]. Further studies will be needed to probe the underlying pathways involved in EED-mediated morphometry disruptions and determine whether these changes are caused by a general response to intestinal inflammation or are a product of more specific mechanisms.

## Author contributions

The authors’ responsibilities were as follows – PBS, SS, CAM, TA, MMa, SAA, PK, SRM: designed research; SS, CM, MSH, LE, CAM, TA, MMa, SAA, PK, SRM: conducted research; DMD, PBS: data management; KV, DC, LE: analyzed data; DMD, KV, PBS: data interpretation; SS, ARG: wrote the paper; DMD, PBS PK: edited manuscript; SS: shared primary responsibility for final content; LE, MFR: generated morphometric measurements; and all authors: read and approved the final manuscript.

## Conflict of interest

The authors report no conflicts of interest.

## Funding

The EEDBI Consortium was funded by the following grants: Bill and Melinda Gates Foundation OPP1136759, OPP1138727, OPP1066118, OPP1144149, and OPP1152812, as well as Advanced Imaging and Tissue Analysis Core, Washington University Digestive Diseases Research Core Center P30DK052574. This work was also supported by an NIH K23 Award through the NIH Institute of Diabetes and Digestive and Kidney Diseases (NIDDK), Award Number 5K23DK117061-034 to Dr. Syed.

## Data availability

Data supporting the findings of this study are available within the article and its supplementary material. Raw data that support the findings of this study are available from the corresponding author, upon reasonable request. Data described in the manuscript, code book, and analytic code will be made available upon request to the corresponding author pending application and approval.

## Figures and Tables

**Table:** 

Surname	Name	Full Affiliation	Email
Ahmed	Kumail	Department of Paediatrics and Child Health, Aga Khan University, Karachi, Pakistan	kumail.ahmed@aku.edu
Ahmed	Sheraz	Department of Paediatrics and Child Health, Aga Khan University, Karachi, Pakistan	sheraz.ahmed@aku.edu
Alam	Md. Ashraful	Nutrition Research Division, International Centre for Diarrhoeal Disease Research, Bangladesh, Dhaka, Bangladesh	mashraful@icddrb.org
Begum	S.M. Khodeza Nahar	Department of Pathology, Bangladesh Specialized Hospital, Dhaka, Bangladesh	khodeza33@hotmail.com
Das	Subhasish	Nutrition Research Division, International Centre for Diarrhoeal Disease Research, Bangladesh, Dhaka, Bangladesh	subhasish.das@icddrb.org
Denson	Lee A.	Division of Pediatric Gastroenterology, Hepatology, and Nutrition, Cincinnati Children's Hospital Medical Center, Cincinnati, OH, USA	Lee.Denson@cchmc.org
Fahim	Shah Mohammad	Nutrition Research Division, International Centre for Diarrhoeal Disease Research, Bangladesh, Dhaka, Bangladesh	mohammad.fahim@icddrb.org
Gazi	Md. Amran	Nutrition Research Division, International Centre for Diarrhoeal Disease Research, Bangladesh, Dhaka, Bangladesh	amran.gazi@icddrb.org
Hasan	Md. Mehedi	Nutrition Research Division, International Centre for Diarrhoeal Disease Research, Bangladesh, Dhaka, Bangladesh	md.hasan@icddrb.org
Hotwani	Aneeta	Department of Paediatrics and Child Health, Aga Khan University, Karachi, Pakistan	aneeta.hotwani@aku.edu
Iqbal	Junaid	Department of Paediatrics and Child Health, Aga Khan University, Karachi, Pakistan	junaid.iqbal@aku.edu
Iqbal	Najeeha Talat	Department of Paediatrics and Child Health, Aga Khan University, Karachi, Pakistan	najeeha.iqbal@aku.edu
Jamil	Zehra	Department of Biological and Biomedical Sciences, Aga Khan University, Karachi, Pakistan	zehra.jamil@aku.edu
Kabir	Furqan	Department of Paediatrics and Child Health, Aga Khan University, Karachi, Pakistan	furqan.kabir@aku.edu
Liu	Ta-Chiang	Department of Pathology and Immunology, Washington University, St. Louis, MO, USA	ta-chiang.liu@wustl.edu
Mazumder	Ramendra Nath	Nutrition Research Division, International Centre for Diarrhoeal Disease Research, Bangladesh, Dhaka, Bangladesh	ramen@icddrb.org
Ragahavan	Shyam S	Department of Pathology, University of Virginia, Charlottesville, VA, USA	SR7WQ@hscmail.mcc.virginia.edu
Rahman	Masudur	Department of Gastroenterology, Sheikh Russel National Gastroliver Institute and Hospital, Dhaka, Bangladesh	drmasud47@yahoo.com
Rahman	Najeeb	Department of Paediatrics and Child Health, Aga Khan University, Karachi, Pakistan	najeeb.rahman@aku.edu
Sadiq	Kamran	Department of Paediatrics and Child Health, Aga Khan University, Karachi, Pakistan	kamran.sadiq@aku.edu
Sarker	Shafiqul Alam	Nutrition Research Division, International Centre for Diarrhoeal Disease Research, Bangladesh, Dhaka, Bangladesh	sasarker@icddrb.org
Tarr	Phillip I.	Department of Pediatrics, Washington University, St. Louis, MO, USA	tarr@wustl.edu
Tearney	Guillermo J.	Department of Pathology, Harvard Medical School, Boston, MA, USA	gtearney@partners.org
Umrani	Fayaz	Department of Paediatrics and Child Health, Aga Khan University, Karachi, Pakistan	fayaz.umrani@aku.edu
Umutesi	Grace	Department of Global Health, University of Washington, Seattle, WA, USA	gumutesi@uw.edu
Yilmaz	Omer H.	Department of Pathology, Massachusetts General Hospital, Boston, MA, USA	oyilmaz@mgh.harvard.edu
