# Supplementary material for: Duodenal quantitative mucosal morphometry in children with environmental enteric dysfunction: a cross-sectional multicountry analysis
Source: Am J Clin Nutr. 2024 Apr 27;120(Suppl 1):S41–50. doi: 10.1016/j.ajcnut.2024.04.027 (PMC11562031; doi:10.1016/j.ajcnut.2024.04.027)
Supplement: Multimedia component 1 [file mmc1.doc]

Supplemental Table 1. List of morphometry, histology, and immunohistochemistry variables used in this study.

| Marker | Function |
| --- | --- |
| Crypt depth | Average depth of crypt based off two measurements. |
| Villus height | Average height of villus based off two measurements. |
| V:C ratio | Ratio of average villus height to average crypt depth. |
| Villus architecture score | Qualitative assessment of villus height in sample  (scored 0-4). |
| Paneth cell depletion | Categorical grouping based on number of Paneth cells per crypt base (scored 0-3). |
| Goblet cell depletion | Categorical grouping based on number of goblet cells per enterocyte (scored 0-4). |
| IEL area | Lymphocyte infiltration – Area of CD3 staining overlapping with KRT18 |
| IEL count | Lymphocyte infiltration – Count of discrete CD3+ cells. |
| CD3 | Immunohistochemical stain for T lymphocytes. |
| KRT18 | Keratin 18 – Intermediate filament cytoskeleton protein. General cell marker. |
| REG1B | Regenerating Family Member 1 Beta – Epithelial crypt cytoplasm; marker of epithelial repair. |
| MKI67 area | Marker Ki67 – Actively proliferating crypt cells. |
| Defensin 5 area | ɑ-defensin peptide 5 – Marker of Paneth cells. |
| SLC15A1 total | Solute carrier family 15 member 1 [PEPT1] – Epithelial brush border marker |
| SLC15A1-b:SLC15A1-c ratio | Area of epithelial brush border (SLC15A1-b) normalized to epithelial cytoplasm (SLC15A1-c) |
| DUOX2 | Dual oxidase 2 – Epithelial cell cytoplasm stain |
| Sucrase isomaltase | Brush border glucosidase enzyme – Functional marker for brush border |

Abbreviations: IEL=intraepithelial lymphocyte, V:C ratio=villus:crypt ratio.

Supplemental Table 2. Linear mixed effects model estimates associating morphometry measurements with histology scores among participants with EED; multivariable results are adjusted for EEDBI center and age.

| Morphometry – Histology Scores | n | Univariate Model  % Difference (95% CI) | Multivariable Model  % Difference (95% CI) |
| --- | --- | --- | --- |
| Villus height – Villus architecture | 64 | -14 (-19, -8) | -13 (-19, -7) |
| Villus height – Paneth cell depletion | 64 | -3 (-11, 5) | -1 (-11, 9) |
| Villus height – Goblet cell depletion | 58 | -4 (-14, 8) | 0 (-13, 14) |
| Crypt Depth – Paneth cell depletion | 69 | -8 (-13, -2) | -4 (-9, 2) |
| V:C ratio – Villus architecture | 58 | -16 (-23, -9) | -18 (-24, -11) |

Morphometry measurements were log-transformed, while histology scores were not. Point estimates are reported as the mean % difference in the morphometry variable per each unit higher histology score. For example: -14 can be interpreted as each 1-unit higher villus architecture score is associated with 14% lower villus height.

Abbreviations: CI=confidence interval, V:C ratio=villus:crypt ratio.

Supplemental Table 3. Linear mixed effects model estimates associating morphometry measurements with immunohistochemistry readouts in EED cohorts, adjusted for EEDBI center and age.

| Morphometry – IHC Variables | Normalization | N | Univariate Model | Multivariable Model |
| --- | --- | --- | --- | --- |
|  |  |  | % Difference (95% CI) | % Difference (95% CI) |
| Villus Height (dependent) |  |  |  |  |
| Lymphocyte infiltration (IEL area) | EA | 46 | 0.9  (-0.8, 2.7) | 0.0  (-1.9, 2.0) |
| Lymphocyte infiltration (IEL count) | EA | 46 | 1.8  (-0.8, 4.4) | 0.8  (-1.8, 3.4) |
| Epithelial brush border (SLC15A1 total) | SA | 41 | 3.0  (1.0, 5.0) | 2.0  (-0.3, 4.4) |
| Epithelial brush border (SLC15A1-b) | SLC15A1-c | 41 | 1.4  (-0.7, 3.6) | 0.5  (-1.6, 2.6) |
| Epithelial cell cytoplasm (DUOX2) | SA | 43 | -0.3  (-0.9, 0.3) | -0.3  (-1.1, 0.5) |
| Brush border (Sucrase isomaltase area) | EA | 46 | 0.3  (-1.5, 2.1) | 0.1  (-1.7, 1.9) |
| Crypt Depth (dependent) |  |  |  |  |
| Lymphocyte infiltration (IEL area) | EA | 46 | 0.8  (-0.2, 1.8) | 0.1  (-0.9, 1.2) |
| Lymphocyte infiltration (IEL count) | EA | 46 | 0.9  (-0.5, 2.4) | 0.3  (-1.2, 1.7) |
| Epithelial repair (REG1B) | SA | 46 | -0.4  (-0.8, 0.0) | -0.4  (-0.8, 0.0) |
| Proliferating crypt cells (MKI67 area) | EA | 44 | 0.6  (-0.4, 1.6) | 0.3  (-1.1, 1.6) |
| Paneth Cell area (Defensin 5) | EA | 46 | -0.2  (-0.7, 0.4) | -0.2  (-0.7, 0.4) |

Morphometry measurements and IHC readouts were log-transformed. Coefficients are reported as the mean % difference in morphometry variable per 10% higher IHC variable. For example: 0.9 can be interpreted as every 10% higher lymphocyte area is associated with a 0.9% higher villus height.

Abbreviations: CI=confidence interval, EA=epithelial area, IEL=intraepithelial lymphocyte, IHC=immunohistochemistry, SA=surface area.
