# Supplementary material for: Duodenal quantitative mucosal morphometry in children with environmental enteric dysfunction: a cross-sectional multicountry analysis
Source: Am J Clin Nutr. 2024 Apr 27;120(Suppl 1):S41–50. doi: 10.1016/j.ajcnut.2024.04.027 (PMC11562031; doi:10.1016/j.ajcnut.2024.04.027)
Supplement: Multimedia component 2 [file mmc2.doc]

Appendix A: The collaborators of the EEDBI Consortium

| **Surname** | **Name** | **Full Affiliation** | **Email** |
| --- | --- | --- | --- |
| Ahmed | Kumail | Department of Paediatrics and Child Health, Aga Khan University, Karachi, Pakistan | [kumail.ahmed@aku.edu](mailto:kumail.ahmed@aku.edu) |
| Ahmed | Sheraz | Department of Paediatrics and Child Health, Aga Khan University, Karachi, Pakistan | [sheraz.ahmed@aku.edu](mailto:sheraz.ahmed@aku.edu) |
| Alam | Md. Ashraful | Nutrition Research Division, International Centre for Diarrhoeal Disease Research, Bangladesh, Dhaka, Bangladesh | [mashraful@icddrb.org](mailto:mashraful@icddrb.org) |
| Begum | S.M. Khodeza Nahar | Department of Pathology, Bangladesh Specialized Hospital, Dhaka, Bangladesh | [khodeza33@hotmail.com](mailto:khodeza33@hotmail.com) |
| Das | Subhasish | Nutrition Research Division, International Centre for Diarrhoeal Disease Research, Bangladesh, Dhaka, Bangladesh | [subhasish.das@icddrb.org](mailto:subhasish.das@icddrb.org) |
| Denson | Lee A. | Division of Pediatric Gastroenterology, Hepatology, and Nutrition, Cincinnati Children's Hospital Medical Center, Cincinnati, OH, USA | [Lee.Denson@cchmc.org](mailto:Lee.Denson@cchmc.org) |
| Fahim | Shah Mohammad | Nutrition Research Division, International Centre for Diarrhoeal Disease Research, Bangladesh, Dhaka, Bangladesh | [mohammad.fahim@icddrb.org](mailto:mohammad.fahim@icddrb.org) |
| Gazi | Md. Amran | Nutrition Research Division, International Centre for Diarrhoeal Disease Research, Bangladesh, Dhaka, Bangladesh | [amran.gazi@icddrb.org](mailto:amran.gazi@icddrb.org) |
| Hasan | Md. Mehedi | Nutrition Research Division, International Centre for Diarrhoeal Disease Research, Bangladesh, Dhaka, Bangladesh | [md.hasan@icddrb.org](mailto:md.hasan@icddrb.org) |
| Hotwani | Aneeta | Department of Paediatrics and Child Health, Aga Khan University, Karachi, Pakistan | [aneeta.hotwani@aku.edu](mailto:aneeta.hotwani@aku.edu) |
| Iqbal | Junaid | Department of Paediatrics and Child Health, Aga Khan University, Karachi, Pakistan | [junaid.iqbal@aku.edu](mailto:junaid.iqbal@aku.edu) |
| Iqbal | Najeeha Talat | Department of Paediatrics and Child Health, Aga Khan University, Karachi, Pakistan | najeeha.iqbal@aku.edu |
| Jamil | Zehra | Department of Biological and Biomedical Sciences, Aga Khan University, Karachi, Pakistan | [zehra.jamil@aku.edu](mailto:zehra.jamil@aku.edu) |
| Kabir | Furqan | Department of Paediatrics and Child Health, Aga Khan University, Karachi, Pakistan | [furqan.kabir@aku.edu](mailto:furqan.kabir@aku.edu) |
| Liu | Ta-Chiang | Department of Pathology and Immunology, Washington University, St. Louis, MO, USA | [ta-chiang.liu@wustl.edu](mailto:ta-chiang.liu@wustl.edu) |
| Mazumder | Ramendra Nath | Nutrition Research Division, International Centre for Diarrhoeal Disease Research, Bangladesh, Dhaka, Bangladesh | [ramen@icddrb.org](mailto:ramen@icddrb.org) |
| Ragahavan | Shyam S | Department of Pathology, University of Virginia, Charlottesville, VA, USA | [SR7WQ@hscmail.mcc.virginia.edu](mailto:SR7WQ@hscmail.mcc.virginia.edu) |
| Rahman | Masudur | Department of Gastroenterology, Sheikh Russel National Gastroliver Institute and Hospital, Dhaka, Bangladesh | [drmasud47@yahoo.com](mailto:drmasud47@yahoo.com) |
| Rahman | Najeeb | Department of Paediatrics and Child Health, Aga Khan University, Karachi, Pakistan | [najeeb.rahman@aku.edu](mailto:najeeb.rahman@aku.edu) |
| Sadiq | Kamran | Department of Paediatrics and Child Health, Aga Khan University, Karachi, Pakistan | [kamran.sadiq@aku.edu](mailto:kamran.sadiq@aku.edu) |
| Sarker | Shafiqul Alam | Nutrition Research Division, International Centre for Diarrhoeal Disease Research, Bangladesh, Dhaka, Bangladesh | [sasarker@icddrb.org](mailto:sasarker@icddrb.org) |
| Tarr | Phillip I. | Department of Pediatrics, Washington University, St. Louis, MO, USA | [tarr@wustl.edu](mailto:tarr@wustl.edu) |
| Tearney | Guillermo J. | Department of Pathology, Harvard Medical School, Boston, MA, USA | [gtearney@partners.org](mailto:gtearney@partners.org) |
| Umrani | Fayaz | Department of Paediatrics and Child Health, Aga Khan University, Karachi, Pakistan | [fayaz.umrani@aku.edu](mailto:fayaz.umrani@aku.edu) |
| Umutesi | Grace | Department of Global Health, University of Washington, Seattle, WA, USA | [gumutesi@uw.edu](mailto:gumutesi@uw.edu) |
| Yilmaz | Omer H. | Department of Pathology, Massachusetts General Hospital, Boston, MA, USA | [oyilmaz@mgh.harvard.edu](mailto:oyilmaz@mgh.harvard.edu) |
